# Perspectives of US Adults on Antimicrobial Trials With Noninferiority Designs

**DOI:** 10.1001/jamanetworkopen.2023.16297

**Published:** 2023-05-31

**Authors:** Robert Morlock, John Rose, John H. Powers

**Affiliations:** 1 Acumen Health Research Institute, Ann Arbor, Michigan; 2 Institute for Transport and Logistics Studies, The University of Sydney Business School, Sydney, New South Wales, Australia; 3 Department of Medicine, George Washington University School of Medicine, Washington, DC

## Abstract

This survey study assesses respondents’ willingness to participate in noninferiority trials of antimicrobials.

## Introduction

New medical interventions are usually studied in trials evaluating improved efficacy vs current standards of care (superiority hypotheses). Increasingly, new medical interventions are studied using noninferiority (NI) hypotheses.^1,2,3^ It is statistically impossible to evaluate whether 2 interventions are identical; therefore, NI trials allow acceptable amounts of prechosen *worse* efficacy with new interventions compared with older, effective interventions, termed the *NI margin*, for patients with effective therapeutic options. NI is ethical when potentially worse efficacy is traded off for other hypothesized advantages (nonefficacy benefits).

International guidance states that NI margins should be acceptable to patients,^4^ yet NI margins are routinely chosen by investigators, pharmaceutical sponsors, and regulatory agencies. Despite calls for patient input on NI trials, little research exists on potential participants’ views of NI trials and margins. This survey study asked US adults about acceptable amounts of worse efficacy and willingness to participate in NI trials of antimicrobials.

## Methods

The study protocol was reviewed by the Sterling institutional review board and classified as exempt because responses were deidentified in accordance with 45 CFR §46. Participants electronically signed informed consent. This study followed the AAPOR reporting guideline.

We performed a cross-sectional, population-based, online survey of US adults participating in a research panel in November 2022. Quotas for age, sex and US Census region were used to support recruitment of a population-based sample. The survey included socioeconomic demographics (eMethods in Supplement 1), behavioral factors, and questions regarding NI trials and acceptable margins of worse efficacy and willingness to participate in NI trials. A sample size of 400 was selected with a margin of error less than ± 5.00%. Results were summarized descriptively. Means and 95% CIs were reported.

## Results

The completion rate of the survey was 83.5%. The analytical sample included 401 adults (mean [SD] age, 48.71 [15.71] years; 205 women [51.1%]; 299 White individuals [74.6%]) (Table). Overall, 358 respondents (89.3%; 95% CI, 86.0%-92.0%) indicated they were concerned antimicrobials were being approved with potentially worse efficacy than approved treatments. A total of 326 respondents (81.3%; 95% CI, 77.3%-84.9%) indicated it would be important to know whether the trials were assessing new antimicrobials using NI designs. When asked to rate on an 11-point scale ranging from 0 (not at all important) to 10 (extremely important) how important it would be to know the NI margin of allowable worse efficacy being tested in the clinical trial, the mean score was 7.8 (95% CI, 7.6-8.0) out of 10. More than one-half of respondents (205 respondents; 51.1%; 95% CI, 46.2%-56.0%) indicated they would not participate in clinical trials testing new antimicrobials allowing 10% worse efficacy compared with already approved alternative interventions. For those indicating they would participate, they would participate if there were other clearly hypothesized benefits offsetting decreased efficacy and if their doctor recommended the trial.

**Table.** Comparison of Study Sample to US Census Population

Characteristic	Individuals, No. (%)	
	US Census 2015-2019 (n = 251 297 494)	Study sample (n = 401)
Sex		
Female	128 932 181 (51.3)	205 (51.1)
Male	122 365 313 (48.7)	196 (48.9)
Age, y		
18-44	116 673 667 (46.6)	184 (45.9)
45-64	83 845 009 (33.4)	128 (31.9)
≥65	50 778 818 (20.2)	89 (22.2)
Race		
Black or African American	30 943 672 (12.3)	59 (14.7)
White	186 270 904 (74.1)	299 (74.6)
Other race^a^	34 082 918 (13.6)	43 (10.7)
Ethnicity		
Hispanic, Latino, or Spanish origin	40 022 823 (15.9)	49 (12.2)
Not Hispanic, Latino, or Spanish origin	211 274 671 (84.1)	352 (87.8)
Region		
Northwest	44 353 930 (17.6)	72 (18.0)
Midwest	52 607 074 (20.9)	80 (20.0)
South	94 998 817 (37.8)	166 (41.4)
West	59 337 673 (23.6)	83 (20.7)

^a^ Other race included 1 or more races (American Indian or Alaska Native, Asian, Native Hawaiian or Pacific Islander) in addition to Black or African American and another race, and White and another race.

## Discussion

This survey study demonstrated that patients want to know the NI margin if they are enrolling in NI trials. More than 50% of respondents would not participate in NI trials where efficacy may be up to 10% worse than existing treatments.

These results have important implications for NI trial design. Recent research showed use of NI hypotheses, NI margins, and trade-offs for nonefficacy benefits are not explained in informed consent of antimicrobial trials;^5^ Our results show this information should be included. Trade-offs for nonefficacy benefits should be clearly stated in consent forms with superiority hypotheses for nonefficacy benefits evaluated in protocols.

Regulatory agencies have allowed up to 10% worse effectiveness for new antimicrobials, increasing to 20% in recent studies.^6^ These findings suggest that NI margins may be unacceptably large, and that smaller NI margins should be required. Further research is needed to assess perceptions of NI margins in specific clinical settings. A limitation of this study is that the results reflect the sample surveyed and are by design representative of the US general adult population.

## Notes

Supplement 1. **eAppendix.** eMethods

Supplement 2.
Data Sharing Statement
