# Supplementary material for: Perspectives of US Adults on Antimicrobial Trials With Noninferiority Designs
Source: JAMA Netw Open. 2023 May 31;6(5):e2316297. doi: 10.1001/jamanetworkopen.2023.16297 (PMC10233412; doi:10.1001/jamanetworkopen.2023.16297)
Supplement: Supplement 1. — eAppendix. eMethods [file jamanetwopen-e2316297-s001.pdf]

Supplemental Online Content

Morlock R, Rose J, Powers JH III. Perspectives of US adults on antimicrobial trials with noninferiority designs. *JAMA Netw Open*. 2023;6(5):e2316297
doi:10.1001/jamanetworkopen.2023.16297

eAppendix. eMethods

This supplemental material has been provided by the authors to give readers additional information about their work.

© 2023 Morlock R et al. *JAMA Network Open.*

eAppendix. eMethods

Race and ethnicity were determined by self-report.Race was categorized as Black or African American; White; or other race (defined as 1 or more races [American Indian or Alaska Native, Asian, Native Hawaiian or Pacific Islander] in addition to Black or African American and another race, and White and another race). Ethnicity was classified as Hispanic, Latino or Spanish origin. Race and ethnicity were included to be reflective of the general US population. Data were analyzed using statistical software SPSS Statistics version 28.0 (IBM).

© 2023 Morlock R et al. *JAMA Network Open.*
