# Supplementary material for: Perspectives of US Adults on Antimicrobial Trials With Noninferiority Designs
Source: JAMA Netw Open. 2023 May 31;6(5):e2316297. doi: 10.1001/jamanetworkopen.2023.16297 (PMC10233412; doi:10.1001/jamanetworkopen.2023.16297)
Supplement: Supplement 2. — Data Sharing Statement [file jamanetwopen-e2316297-s002.pdf]

Data Sharing Statement

Morlock. Perspectives of US Adults on Antimicrobial Trials With Noninferiority Designs. *JAMA Netw Open*. Published May 31, 2023. doi:10.1001/jamanetworkopen.2023.16297

Data
Data available: Yes
Data types: Deidentified participant data
How to access data: Data will be made available to address pre-specified research questions, to researchers whose proposed use of the data has been approved, and with whom there is a signed data access agreement.
When available: With publication

Supporting Documents
Document types: None

Additional Information
Who can access the data: Data will be made available to address pre-specified research questions, to researchers whose proposed use of the data has been approved, and with whom there is a signed data access agreement.
Types of analyses: Data will be made available to address pre-specified research questions, to researchers whose proposed use of the data has been approved, and with whom there is a signed data access agreement.
Mechanisms of data availability: Data will be made available to address pre-specified research questions, to researchers whose proposed use of the data has been approved, and with whom there is a signed data access agreement.
